# Unveiling metabolic pathways of selected plant-derived glycans by *Bifidobacterium pseudocatenulatum*

**DOI:** 10.3389/fmicb.2024.1414471

**Published:** 2024-07-16

**Authors:** Rocio Sanchez-Gallardo, Francesca Bottacini, Lisa Friess, Maria Esteban-Torres, Clarissa Somers, Rebecca L. Moore, Fionnuala M. McAuliffe, Paul D. Cotter, Douwe van Sinderen

**Affiliations:** ^1^APC Microbiome Ireland, University College Cork, Cork, Ireland; ^2^School of Microbiology, University College Cork, Cork, Ireland; ^3^Department of Biological Sciences, Munster Technological University, Cork, Ireland; ^4^UCD Perinatal Research Centre, School of Medicine, National Maternity Hospital, University College Dublin, Dublin, Ireland; ^5^Teagasc Food Research Centre Moorepark, Cork, Ireland

**Keywords:** plant carbohydrates, gut microbiota, starch, xylan, bifidobacteria

## Abstract

Bifidobacteria are commonly encountered members of the human gut microbiota that possess the enzymatic machinery necessary for the metabolism of certain plant-derived, complex carbohydrates. In the current study we describe differential growth profiles elicited by a panel of 21 newly isolated *Bifidobacterium pseudocatenulatum* strains on various plant-derived glycans. Using a combination of gene-trait matching and comparative genome analysis, we identified two distinct xylanases responsible for the degradation of xylan. Furthermore, three distinct extracellular α-amylases were shown to be involved in starch degradation by certain strains of *B. pseudocatenulatum*. Biochemical characterization showed that all three α-amylases can cleave the related substrates amylose, amylopectin, maltodextrin, glycogen and starch. The genes encoding these enzymes are variably found in the species *B. pseudocatenulatum*, therefore constituting a strain-specific adaptation to the gut environment as these glycans constitute common plant-derived carbohydrates present in the human diet. Overall, our study provides insights into the metabolism of these common dietary carbohydrates by a human-derived bifidobacterial species.

## Introduction

Plant-derived carbohydrates typically represent complex polysaccharides present in the cell wall of cereals, fruits, legumes and vegetables, and thus are common components of the human diet. These dietary complex carbohydrates, often referred to as dietary fibres, generally cannot be (or are very poorly) degraded by the human digestive system, except for starch that can be partially degraded by host enzymes and thus incompletely reaches the gut (Kelly et al., 2021). They can however serve as metabolic substrates for saccharolytic components of the human gut microbiota and may act as prebiotics by modulating gut microbiota composition, diversity and metabolic activity (Cronin et al., 2021).

Members of the *Bifidobacterium* genus are among those bacterial species that are able to directly or indirectly ferment some of these complex dietary carbohydrates, mainly releasing acetic acid and lactic acid as metabolic end products, with purported beneficial impact on human health (Pokusaeva et al., 2011; Usta-Gorgun and Yilmaz-Ersan, 2020). Indeed, up to 13% of the bifidobacterial genome content is dedicated to carbohydrate metabolism, highlighting the importance of getting access to a wide range of carbohydrates available in the gut (Kelly et al., 2021). Bifidobacterial species possess varying abilities with respect to carbohydrate metabolism, with some species such as *B. longum* subsp. *longum* and *B. adolescentis* being highly adapted to grow on plant-derived carbohydrates present in the adult diet, while strains *B. longum* subsp. *infantis* and *B. bifidum* being specifically adapted to metabolise sugars present in the infant gut, in particular human milk oligosaccharides (Turroni et al., 2009; Arboleya et al., 2016).

Bifidobacterial degradation of dietary polysaccharides typically requires extracellular, cell envelope-associated enzymes which cleave such glycans into oligosaccharides. These oligosaccharides are then transported intracellularly, where they are further hydrolysed to monosaccharides before they enter the so-called bifid shunt (O’Connell Motherway et al., 2008; Pokusaeva et al., 2011). However, the extracellularly released oligosaccharides may also benefit other gut commensals as it may allow cross-feeding with a consequent impact on gut microbiota composition (Turroni et al., 2018; Culp and Goodman, 2023).

The genomes of human-derived bifidobacteria encode a diverse set of glycosyl hydrolases (GHs), which are responsible for hydrolysis of poly- and oligosaccharides (O’Callaghan and van Sinderen, 2016). As mentioned above, gut commensal-mediated degradation of polymeric glycans, such as starch, galactan, xylan or arabinoxylan, requires extracellular GHs with either exo- or endo-hydrolytic activity (Saito et al., 2020; Deehan et al., 2022). Starch, which represents the main carbohydrate present in a plant-based diet, is a polysaccharide made up of amylose, which consists of a straight chain of α-1,4-connected glucose moieties, and amylopectin, which has in addition to the straight chain α-1,6-branched side chains of α-1,4-maltooligosaccharides (Duranti et al., 2014). In the case of amylose or amylopectin an α-amylase (a GH13 family member) is needed to cleave the large substrate into malto-oligosaccharides, whereas a xylanase (a GH10 family member) is necessary to cleave the xylan backbone consisting of xylose moieties linked together by β-1,4-glycosidic bonds, into xylo-oligosaccharides (XOS) (Viborg et al., 2013; Jung et al., 2020; Watanabe et al., 2021). However, xylan is often found in nature as part of more complex structures, such as arabinoxylan, which requires additional enzymatic activities for degradation (Komeno et al., 2022).

Previous studies have shown that *B. breve* UCC2003 can metabolize starch due to its ability to produce an extracellular type II amylopullulanase, denoted ApuB, which possesses two catalytic domains. The N-terminal portion of ApuB incorporates an α-1-4 amylase domain which facilitates hydrolysis of α-1-4-glucosidic linkages, while a pullulanase domain is present in the C-terminal portion of ApuB, allowing hydrolysis of α-1-6-glucosidic linkages (O’Connell Motherway et al., 2008). Members of the *B. adolescentis* taxon have also been reported to utilize starch and related/derived glycans, such as maltose, maltodextrin and amylopectin, and this species has even been described as being able to colonise starch granules (Duranti et al., 2016; Jung et al., 2020). Two genes, predicted to encode α-glucosidases, have been postulated to be responsible for starch degradation by this species, although biochemical data to support this notion is currently lacking (Duranti et al., 2014). Based on previous studies, *B. longum* subsp. *longum* is usually unable to utilize starch, yet is able to metabolize smaller starch-derived oligosaccharides, such as malto-oligosaccharides and maltodextrin, and may thus access such substrates through cross-feeding based on activities by starch-degrading bacteria such as *Bacteroides* spp. or other bifidobacterial species (Ryan et al., 2006; Liu et al., 2015; Arboleya et al., 2018; Nishiyama et al., 2021). *Bifidobacterium pseudocatenulatum* has been reported to utilize starch, although no further genomic investigations have been carried out to identify the genes involved in starch metabolism in this species (Baruzzi et al., 2017).

Several recent studies have highlighted the apparently unique ability (among bifidobacteria) of *B. pseudocatenulatum* to metabolize xylan and the ability of this glycan to modulate the gut microbiota not only in a pig model but also in humans (Wang et al., 2021; Watanabe et al., 2021; Drey et al., 2022). Watanabe and colleagues identified and characterized a cell envelope-associated/extracellular β-1,4-endoxylanase belonging to the GH10 family responsible for extracellular xylan degradation into XOS (Wang et al., 2021).

The current study provides information regarding the utilization of several plant-derived glycans, in particular starch and derivatives and xylan by a set of recently isolated human-derived *B. pseudocatenulatum* strains. We identify key genes involved and describe the deduced metabolic pathways involved in starch and xylan degradation employed by this species.

## Results

### Growth profiles of *B. pseudocatenulatum* strains cultivated in various plant-derived carbohydrates

In a previous study, we described the isolation of twenty-one genetically distinct *B. pseudocatenulatum* strains from faecal samples obtained from mother-infant dyads (Feehily et al., 2023; Moore et al., 2023). In order to investigate if these isolates are able to metabolize plant-derived glycans, their growth ability was assessed in modified MRS (mMRS) medium supplemented with 0.5% (w/vol) of a given plant carbohydrate as the sole carbon source.

The carbohydrate substrates used were divided into two sets: starch and related sugars, and hemicellulose-associated plant carbohydrates. The first set included: amylopectin, glucose, maltose, maltodextrin, glycogen, pullulan and starch, all of which were assessed as growth substrates following incubation at 37°C for 10 h. The second set included arabinose, galactan (lupin), galactan (potato), arabinogalactan, wheat arabinoxylan and xylan, which were assessed as growth substrates following incubation at 37°C for 24 h.

**Figure 1:**
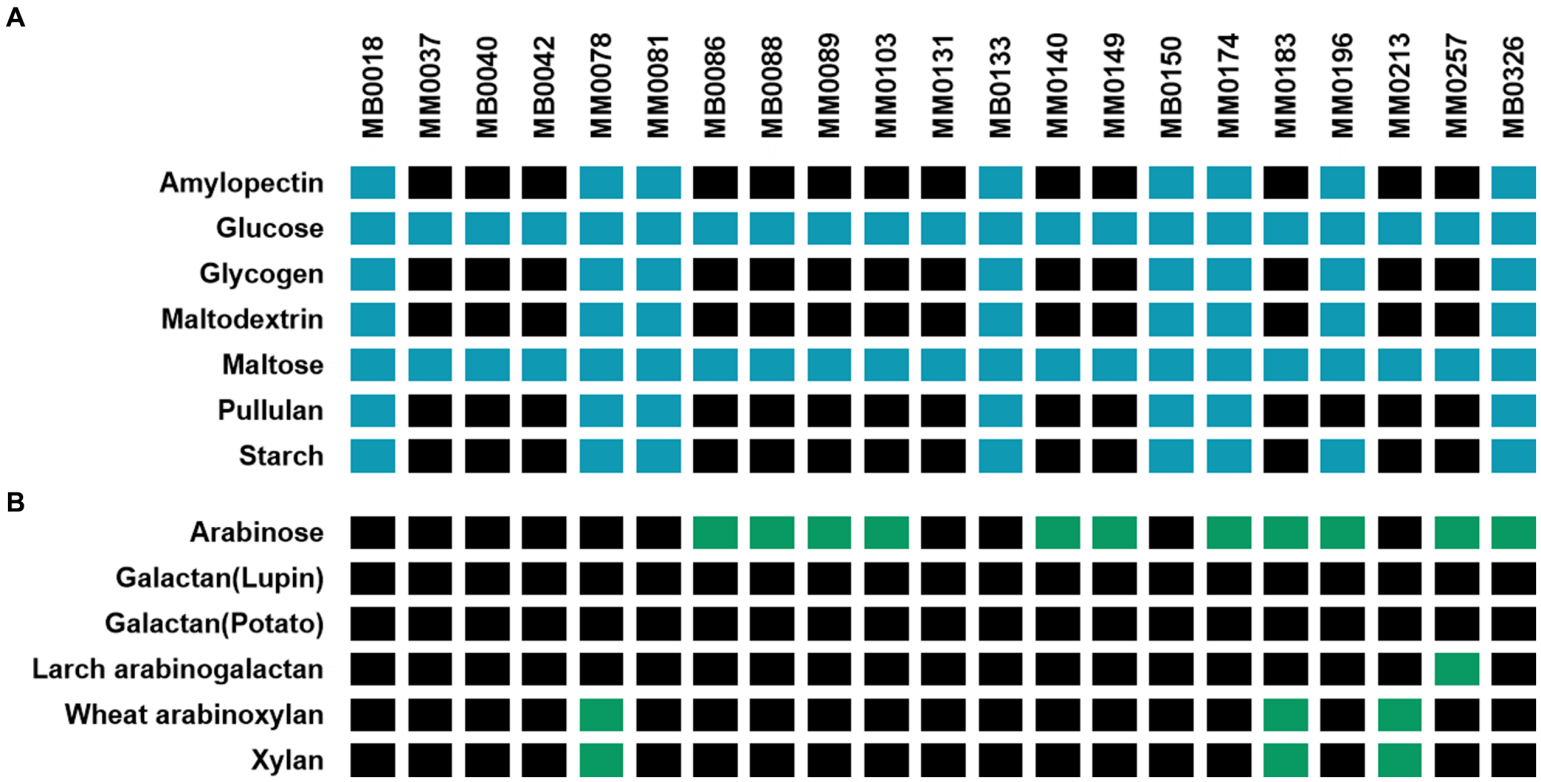
Schematic heatmap showing the growth performance of various *B. pseudocatenulatum* strains (names are indicated at the top of the panels) on different plant-derived carbohydrates used as carbon sources (indicated on the left-hand margin of the panels). A strain was considered to be able to ferment a given carbohydrate when an OD_600nm_ above 0.35 was obtained after incubation (for two biological replicates). **(A)** Growth abilities on glucose and α-linked glucose-containing oligo/polymers (Blue: growth / Black: non-growth) assessed after 24 h of incubation. **(B)** Growth abilities on various, non-glucose-containing plant-derived carbohydrates (Green: growth / Black: non-growth) assessed after 10 h of incubation. Amylose was excluded from this test due to solubility problems in the growth medium used.

As displayed in Figure 1, all tested *B. pseudocatenulatum* strains were shown to grow in glucose and maltose. Interestingly, strains that are able to utilize starch, i.e., MB0018, MM0078, MM0081, MB0133, MB0150, MM0174, MB0326 and MB0196, were also shown to utilize related carbohydrates (glycogen, amylopectin and pullulan, with just a single exception: strain MB0196 is unable to grow on pullulan). Conversely, strains that did not exhibit appreciable growth on starch as the sole carbohydrate source (i.e., strains MM0034, MM0037, MB0040, MB0042, MB0086, MB0088, MM0089, MM0103, MM0131, MM0140, MM0149, MM0183, MM0213, MM0257 and MM0327), were also unable to grow on glycogen, amylopectin or pullulan.

Only a small number of *B. pseudocatenulatum* strains were shown to be able to utilize the carbohydrates from the second set of tested carbohydrates (Figure 1B). Eleven strains were able to grow on arabinose as the sole carbohydrate source and, of these, one strain can also utilize wheat arabinoxylan. Strains MM0078, MM0183 and MM0213 were demonstrated to grow on xylan. Galactose-containing polysaccharides were not utilized by *B. pseudocatenulatum*, except for strain MM0257 which was shown to utilize Larch arabinogalactan as a growth substrate.

### *In silico* analysis of the xylan utilization cluster of *B. pseudocatenulatum* strains

**Figure 2:**
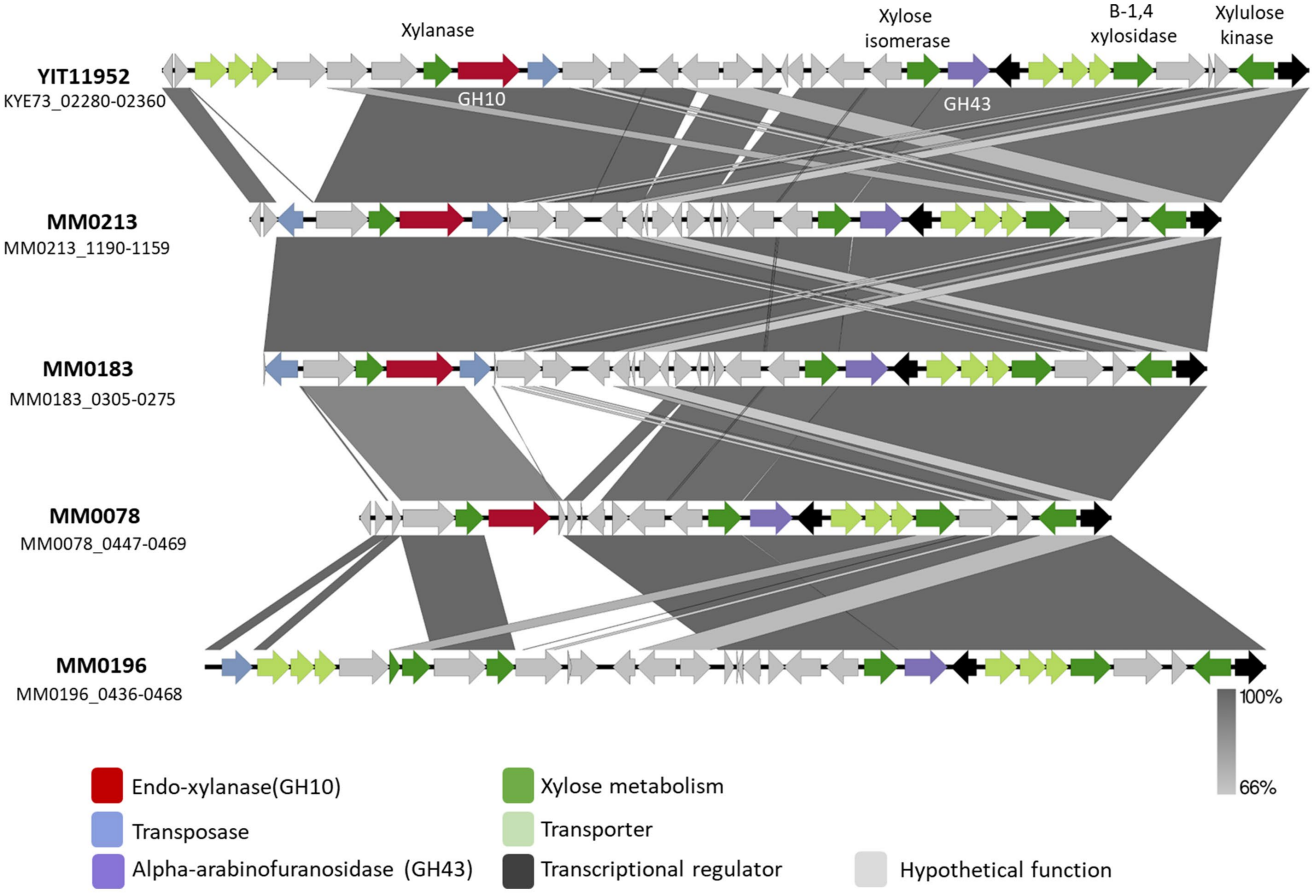
Schematic representation of the *xyl* gene cluster identified in *B. pseudocatenulatum* strains YIT11952, MM0213, MM0183, MM0078 and MM0196. Predicted functions are represented in different colour according to the legend. Percentage of identity elicit in grey scale colours based on blastn.

Xylan is a polysaccharide made up of β-1,4-linked xylose moieties, and this glycan can be metabolized by certain *B. pseudocatenulatum* strains due to the activity of an extracellular endoxylanase belonging to the GH10 family (Watanabe et al., 2021). Watanabe and colleagues identified the gene *BpXyl*10A encoding this key enzyme for xylan metabolism by transcriptome analysis, and described an associated xylan-induced gene cluster (here referred to as *xyl* for xylan utilization), of which some encoded proteins were predicted to be involved in xylan utilization, such as an ABC-type carbohydrate transporter, and several distinct β-xylosidases (Figure 2) (Watanabe et al., 2021).

An initial *in silico* analysis carried out on the genomes employed in this study allowed the identification of a GH10 family member encoded by each of three *B. pseudocatenulatum*, i.e., MM0078, MM0183 and MM0213, being consistent with their ability to grow in a medium with xylan as the sole carbon source (Figure 1). When the DNA sequence of these three genes was compared with that described by Watanabe et al., it was observed that the GH10-encoding genes of strains MM0213 and MM0183, designated here as *xylA_213_ and xylA_183_*, respectively, are nearly identical to each other and to that described by Watanabe et al., sharing more than 99% sequence identity. The GH10-encoding gene present in MM0078, designated here as *xylB_78_*, elicits a substantially lower identity percentage (70.43%). In all three cases, the deduced amino acid sequences of XylA_213_, XylA_183_ and XylB_78_ were predicted to contain a secretion signal, indicating that these proteins represent extracellular enzymes. Furthermore, since the XylA_213/183_ and XylB_78_ proteins are also predicted to contain a C-terminal transmembrane domain, they may stay attached to the cell envelope following secretion.

Interestingly, when compared to the *xyl* gene cluster of *B. pseudocatenulatum* YIT11952, the genetic composition of the *xyl* cluster found in MM0078, MM0213 and MM0183 differs in each of these xylan-utilizing strains (Figure 2). As mentioned above, all of these four xylan-utilizing strains contain a predicted extracellular GH10-encoding gene, while those that do not, for example MM0196, lack such a gene (Figure 2). However, the latter strain does contain many other genes of the *xyl* gene cluster, including those with a predicted involvement in xylose or xylo-oligosaccharide degradation. The main genomic difference observed between the *xyl* cluster present in strains MM0213 and MM0078 is the absence of 7 genes whose function is mostly hypothetical except for one predicted xylulose kinase and a XylR-type transcriptional regulator corresponding to locus tags MM0213_1177–1,178. The *B. pseudocatenulatum* YIT11952 *xyl* cluster possesses a genetic organization that differs from other assessed strains, possessing a region of 5 genes at the beginning of the cluster as well as two additional genes, most of which are hypothetical proteins (Figure 2).

### Biochemical characterization of the GH10 family xylanases encoded by *B. pseudocatenulatum* MM0078 and MM0213

**Figure 3:**
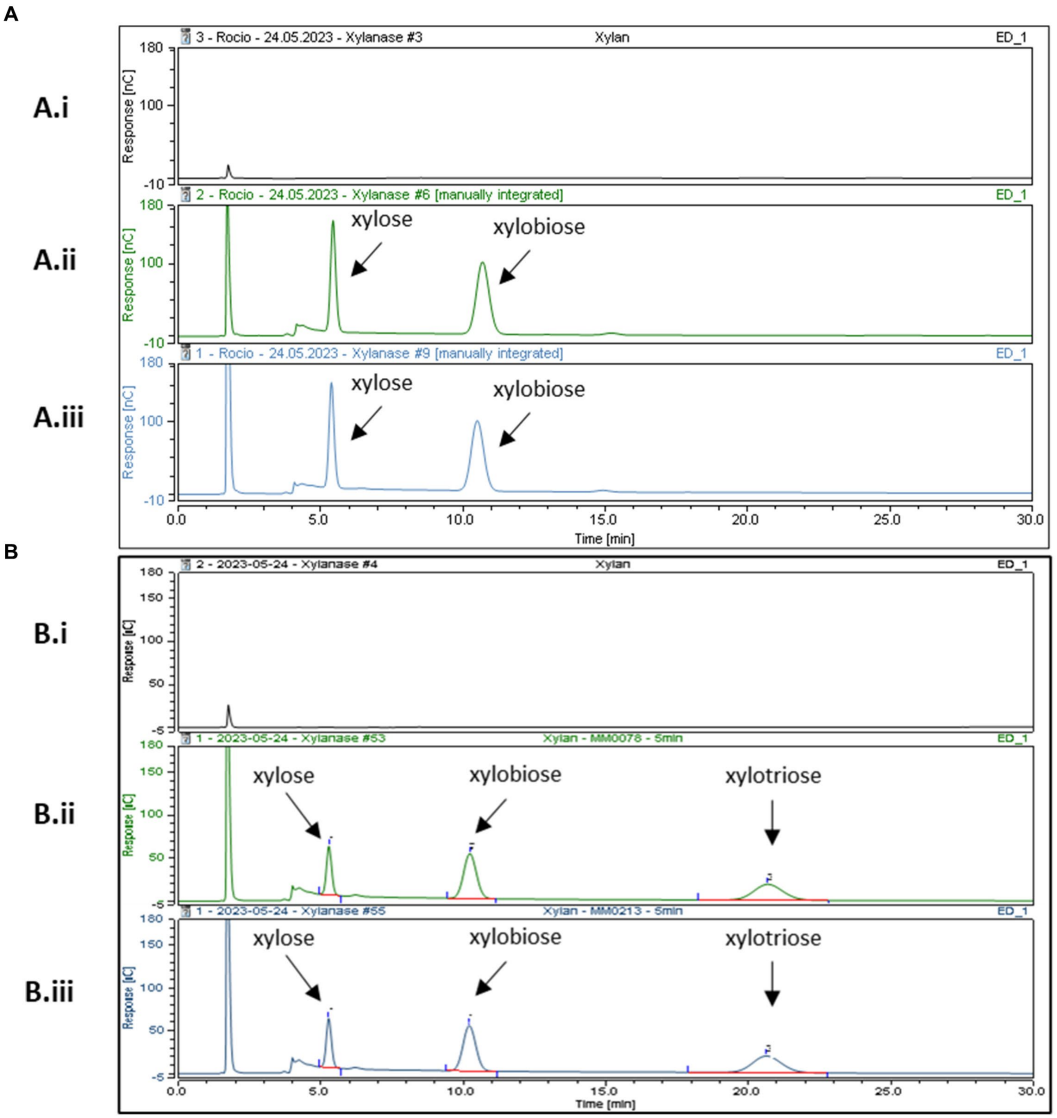
HPAEC-PAD chromatogram profiles after incubation at 37°C in xylan. Panel **(A)** After 16 h of incubation (Ai) xylan incubated with buffer (Aii) XylB _His_ (Aiii) XylA_His_. Panel **(B)** After 5 min of incubation (Bi) xylan in buffer, (Bii) XylB _His_ and (Biii) XylA_His_. Reaction carried out in MOPS buffer pH 7.

In order to further characterize the predicted GH10 family xylanases encoded by *xylB_78_* and *xylA_213_* of *B. pseudocatenulatum* strains MM0078 and MM0213, respectively, these two genes were individually cloned into the IPTG-inducible expression vector pET28b without their secretion signal and transmembrane regions located at their 5′- and 3′-end, respectively, and with the incorporation of a His_10_-encoding tag at their 5′-end, to facilitate overexpression and purification of the respective proteins XylA_213His_ and XylB_78His_ using the *E. coli* protein expression host BL21 (see Materials and Methods for details). Purified XylA_213His_ and XylB_78His_ were then employed for enzymatic reactions using xylan as a substrate. Purified XylA_213His_ and XylB_78His_ were indeed shown to cleave xylan, releasing xylose and xylobiose as products of the reaction following 16 h incubation when the reaction products were analyzed by HPAED-PAD (Figure 3A).

Previous results from Watanabe and colleagues reported that recombinant purified BpXyn10A(XylA) was able to cleave Xylan from Oat Spelt and Beechwood releasing different XOS fractions, including xylobiose, xylotriose, xylotetraose, xylopentaose and xylohexaose. Their reactions were assessed after 2, 8, and 24 h and analyzed using thin-layer chromatography. It is interesting to note that these authors also observed that the amount of released xylose and xylobiose as a product of the reaction increased over time. However, XOS fractions representing those with a higher degree of polymerization, such as xylohexaose, were reduced with increasing incubation times. Extended incubation times thus lead to relatively small products, perhaps explaining why in our experimental set-up we mainly observed two products (xylose and xylobiose) by HPAEC-PAD detection (Figure 3).

To investigate if XylB_78His_ and XylA_213His_ exhibit differential enzymatic characteristics, their activity with respect to xylan was assessed at different incubation times: 5, 15, 30 min, 1, 2, 4 and 6 h. No major differences were observed although xylotriose was produced in the reactions by both enzymes at these shorter incubation times. This product disappeared in the case of XylB_78His_ at incubations longer than 5 min, while for XylA_213His_ this product was still detected at 4 h (Figures 3B,C). These findings suggest that XylA_His_ degrades its substrate at a slightly lower rate, though this may also be due to the recombinant nature of the two enzymes (His-tagged and their native N- and C-terminal sequences removed) or by a different ratio between the endo- and exo-glycosidic activities of the enzymes. Further studies employing a broader range of substrates, such as different arabinoxylans, will be needed to assess possible differences in substrate specificity. Overall, we validate the expected xylanase activity of XylA_213His_ and demonstrate that the similar XylB_78His_ also exhibits xylanase activity, hereby describing a novel endo-xylanase present in the *B. pseudocatenulatum* taxon.

### Transcriptome analysis of *B. pseudocatenulatum* MM0196 in response to starch utilization

As observed above, only eight out of the 21 assessed *B. pseudocatenulatum* strains were shown to utilize starch and other glycans with similar glucosidic linkages, suggesting that these strains encode an α-amylase activity required to degrade such substrates. Therefore, in order to identify the expected genes involved in starch degradation in *B. pseudocatenulatum* MM0196, a transcriptomic analysis was undertaken to identify genes whose transcription was upregulated when this strain was cultivated in a medium containing starch as the sole carbon source (and when compared to a lactose grown control).

Subsequent data analysis revealed a total of 165 differentially expressed genes (DEGs), consisting of 68 up-regulated and 117 down-regulated genes, when the transcriptome of strain MM0196 cultivated in a starch-containing medium was compared to that obtained for lactose-mediated growth. Among the upregulated genes, we identified three gene clusters with distinctly different predicted functions. One of these genomic regions encompasses the genes with locus tags MM0196_0074 through to MM0196_0078, which are predicted to be responsible for the biosynthesis of sortase-dependent pili by this bacterium. It is interesting to note that a transcriptome study involving *B. adolescentis* 22 L reported that transcription of a putative pilus-biosynthesis gene cluster is up-regulated when this strain was cultivated in starch (Duranti et al., 2014). Pilus production by a gut commensal may indeed facilitate gut colonisation as has been shown previously (Turroni et al., 2013), and perhaps a common dietary glycan such as starch may provide a niche-specific signal to produce such extracellular (adhesion) structures. The second starch-induced genomic region corresponds to a prophage-like element previously identified in this genome, although its role, if any, in starch metabolism is unclear (Mavrich et al., 2018).

The third genomic region whose transcription is upregulated when strain MM0196 is grown on starch corresponds to a cluster of ten genes, referred to here as the *amy* cluster, with locus tags MM0196_1449 through to MM0196_1458, which includes two genes on either flank of the cluster, each encoding a predicted GH13-family member with α-amylase activity, and designated here as *amy1* (corresponding to locus tag MM0196_1449) and *amy2* (corresponding to locus tag MM0196_1458). Despite differing in sequence and size, they both are predicted as extracellular α-amylases (Table 1). Of the ten genes of the *amy* cluster of strain MM0196, eight were shown to be transcriptionally upregulated upon cultivation of the strain in starch. As mentioned above, two putative α-amylase-encoding genes flank this gene cluster on either side, while the remaining eight genes of the *amy* cluster are predicted to encode the biosynthetic machinery for an extracellular polysaccharide (EPS; see Table 1). The significance of possible EPS production upon growth in starch is not immediately clear, although EPS has been shown to facilitate long-term colonization, support the intestinal barrier and modulate the host immune response (Fanning et al., 2012; Bottacini et al., 2014).

**Table 1:** *B. pseudocatenulatum* MM0196 genes upregulated in expression during growth in mMRS medium supplemented with 0.5% starch or lactose as the sole carbohydrate.

Locus tag	log2FoldChange	*p*-value	Annotation
MM0196_1449	2.561142	1.01E-236	**a-amylase (GH13 family)**
MM0196_1450	2.477997	2.07E-58	Polysaccharide pyruvyl transferase
MM0196_1451	2.376682	5.02E-32	Glycosyl transferase family 2
MM0196_1454	2.863399	1.10E-42	Flippase, peptidoglycan synthesis
MM0196_1455	3.177669	3.47E-64	Glycosyl transferase
MM0196_1456	4.065458	1.20E-96	Glycosyl transferase family 2
MM0196_1457	3.572252	3.96E-147	Glycosyl transferase
MM0196_1458	2.109952	4.98E-292	**a-amylase (GH13 family)**

^*^The level of expression is shown as a fold-value of increase in expression on starch to a lactose control, with a cut-off of a minimum 2-fold increase in expression. The functions of the genes focused on in this study are shown in bold script. The cutoff point is 2-fold, with a *p* value of < 0.001. Complete data set can be found in Supplementary material.

**Figure 4:**
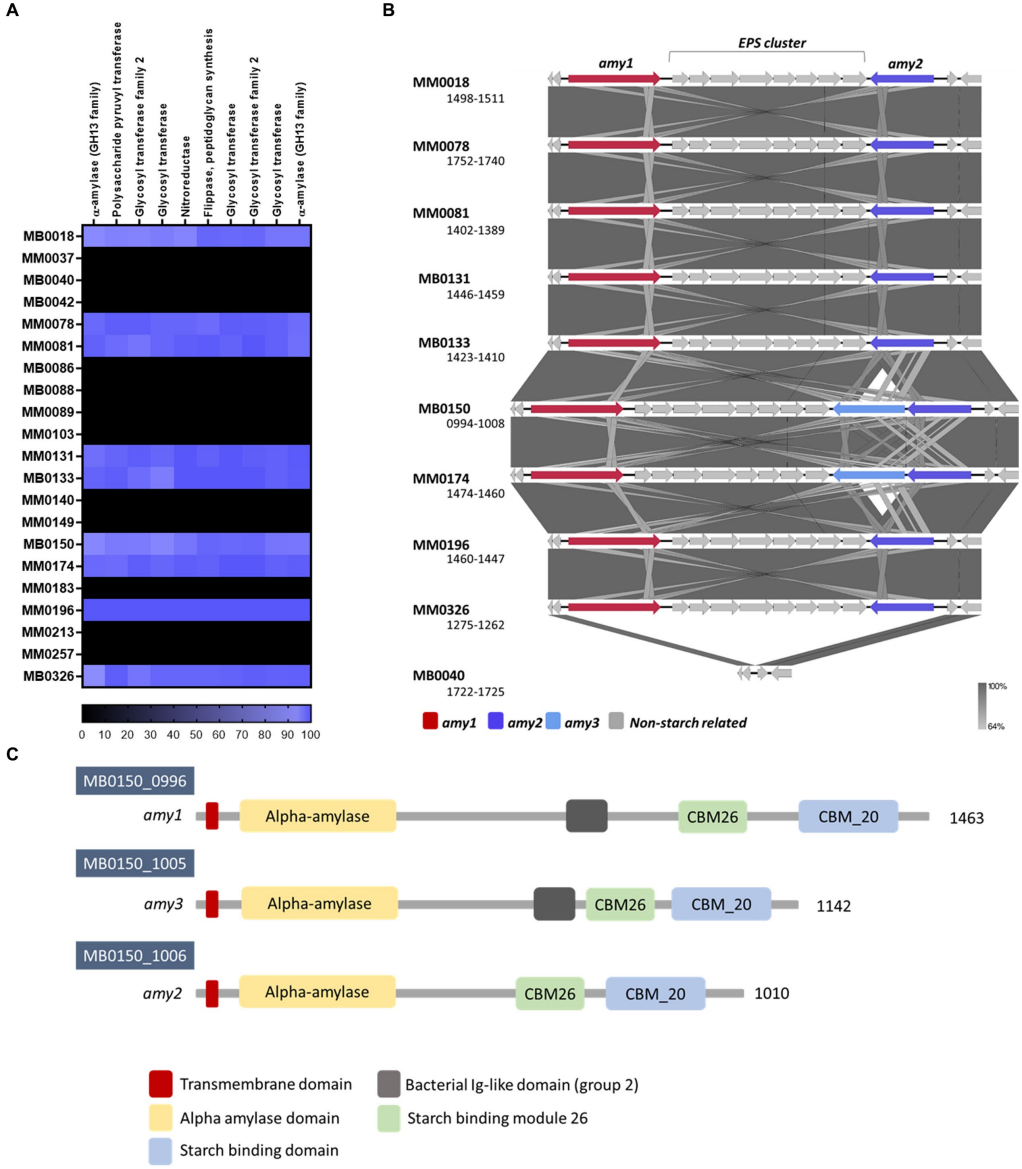
**(A)** Heatmap representing the percentage identity of the genes of the *amy* cluster among the *B. pseudocatenulatum* strains used in this study. **(B)** Genetic representation of the *amy* locus in various *B. pseudocatenulatum* strains assessed in this study. Arrows represent genes and the grey-scale legend underneath indicates identity levels among the *amy* loci as based on blastn. Strain MB0040 was included as a representative lacking this genomic region and being unable to grow on starch. **(C)** Domains of the three predicted α-amylases encoded by *B. pseudocatenulatum* MB0150. Numbers indicated at the right-hand side of each gene representation correspond to the number of amino acid residues of each protein. Predictions performed by HHpred, HMMER and Pfam.

In order to investigate if the presence of the *amy* gene cluster of strain MM0196 correlates with the phenotypic data as described above, a comparative analysis was carried out to identify any homologous genes across the *B. pseudocatenulatum* genomes employed in this study. Figure 4A displays a compilation of the homologous genes present in relevant genomes of this study and shows that eight strains possess a gene cluster highly homologous to the *amy* cluster present in strain MM0196 where all the genes have a percentage identity higher than 98%. Interestingly, the presence of this cluster is correlated with their ability to grow in starch, except for strain MM0131. The inability of MM0131 in this regard may be due to point mutations in *amy1*/*amy2*, lack of expression, or a defective uptake system of maltooligosaccharides that would prevent the strain from growing despite having two functional extracellular α-amylases. Also notable is the presence of a third putative α-amylase-encoding gene, designated here as *amy3*, in the *amy* clusters of strains MB0150 and MM0174 (Figure 4B). As observed in Figure 4B. *B. pseudocatenulatum* MB0040, which is a strain unable to grow on starch, lacks the *amy* locus.

An alignment confirmed that *amy1*, *amy2* and *amy3* from strain MM0150 are different from each other, despite having the same predicted function. Amy1 is the largest and the most distinct of the three α-amylases. The percentage identity between the deducted amino acid sequence of Amy1 and Amy2 is 66.67% with a 98% coverage; while it is 48.30% between Amy 1 and Amy3 with a 93% coverage. Amy2 and Amy3 are highly similar at 96.27%, though they are distinct in size (Figure 4). All three proteins possess an α-amylase domain, as well as two starch-binding domains (CBM20 and CBM26), predicted to be necessary to recognize starch while the α-amylase domain is presumed to be responsible for cleaving the glucosidic linkages. Their catalytic site is located between the residues 171 and 319 for Amy1, between 88 to 270 amino acids for Amy2 and between 84 to 342 for Amy3. However, Amy1 and Amy3 both contain a fourth domain which is predicted to represent a bacterial Ig-like domain, which may be involved in carbohydrate recognition and can play an important role in cell adhesion, influencing bacterial host-cell interaction (Figure 4C) (Kelly et al., 1999; Sidar et al., 2020).

### Biochemical characterization of amy1, amy2 and amy3

**Figure 5:**
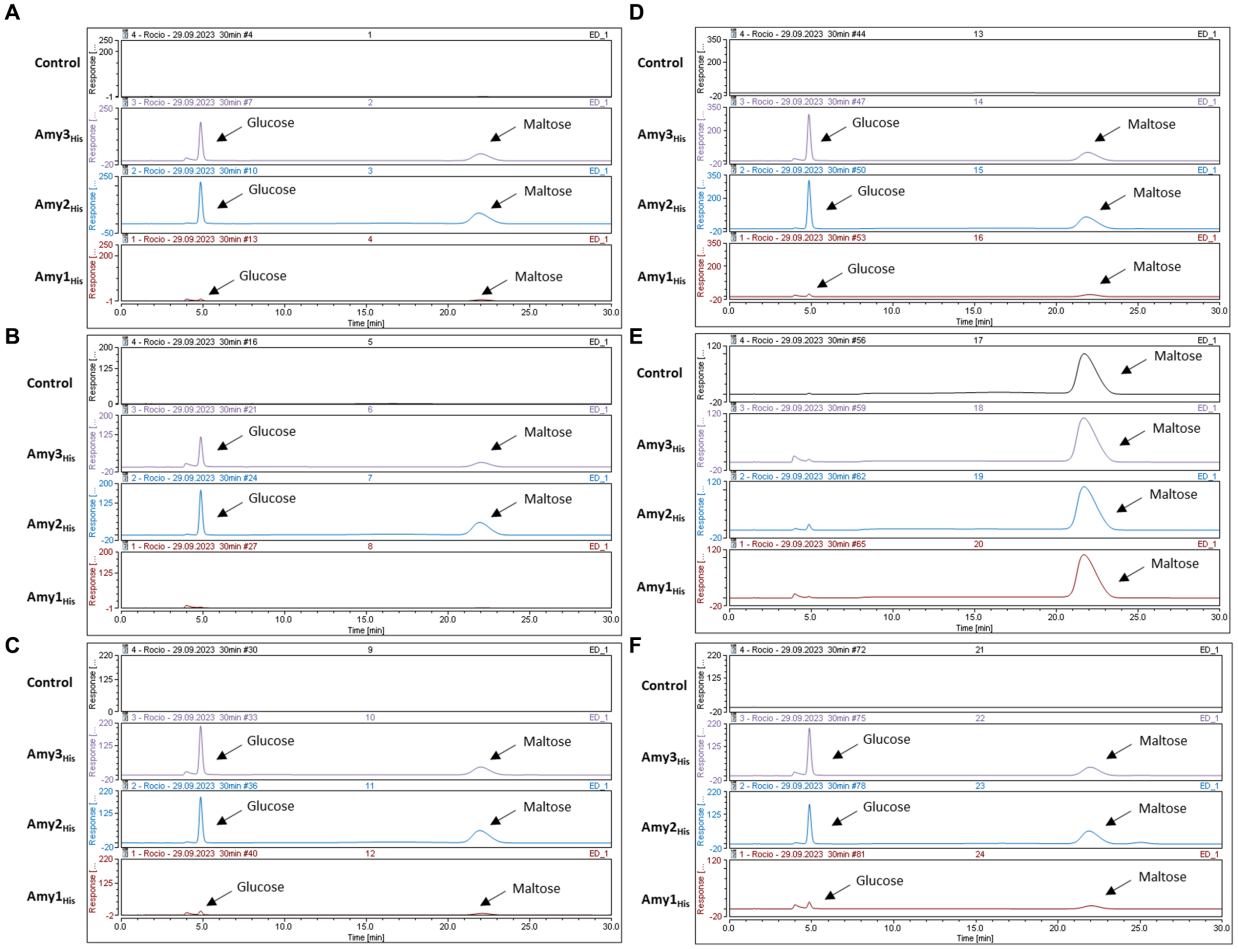
HPAEC-PAD chromatograms profiles after 24 h incubation at 37C with different substrates. **(A)** Starch (Control) with the product of Amy3_His_, Amy2_His_ and Amy1_His_. **(B)** Amylopectin (Control) with the product of Amy3_His_, Amy2_His_ and Amy1_His_. **(C)** Amylose (Control) with the product of Amy3_His_, Amy2_His_ and Amy1_His_. **(D)** Maltodextrin (Control) with the product of Amy3_His_, Amy2_His_ and Amy1_His_. **(E)** Maltose (Control) with the product of Amy3_His_, Amy2_His_ and Amy1_His_. **(F)** Glycogen (Control) with the product of Amy3_His_, Amy2_His_ and Amy1_His_.

Transcriptomic and comparative genomic analysis facilitated the identification of up to three distinct, putative α-amylases encoded by certain *B. pseudocatenulatum* strains, presumably allowing these strains to grow on starch and related carbohydrates, such as amylopectin, maltodextrin and glycogen. The *B. pseudocatenulatum* MB0150 gene products of *amy1_150_*, *amy3_150_* and *amy2_150_* were expressed in *E. coli* excluding their secretion signal and incorporating a His-tag in order to investigate their enzymatic activity and confirm their predicted function. The resulting products of these trimmed and His-tagged *amy1_150_*, *amy2_150_* and *amy3_150_* genes were purified and the obtained recombinant proteins, designated Amy1_His_, Amy2_His_ and Amy3_His_, were incubated with various potential substrates (i.e., starch, amylose, amylopectin, maltodextrin, maltose, glycogen and pullulan). The products of these reactions were then analysed using HPAEC-PAD (Figures 5A-D, F).

Amy2_His_ and Amy3_His_ were shown to cleave starch, amylose, amylopectin, maltodextrin and glycogen, releasing glucose and maltose as a result of their enzymatic activity, confirming their predicted α-amylase activity. Interestingly, none of the proteins was able to act on maltose, suggesting that only larger oligosaccharides can serve as substrates for these enzymes in order to cleave their glucosidic linkages (Figure 5E). Pullulan was not cleaved by any of the proteins, confirming that none of the tested enzymes elicits pullulanase activity, unlike the orthologous ApuB enzyme encoded by *B. breve* UCC2003 (O’Connell Motherway et al., 2008) (Supplementary Figure 1A). Amy1_His_ was shown to degrade starch, amylose, maltodextrin and glycogen, releasing glucose and maltose to some extent as a result of its enzymatic activity. However, this was only observable at a lower scale (Supplementary Figure 2B). Poor production of this recombinant protein may have hampered its enzymatic activity, perhaps due to the size of the protein (as it is substantially larger than Amy2 and Amy3) or because of misfolding as a result of the removal of the signal peptide and the incorporation of the His-tag.

Despite using the same initial concentration of substrate and enzyme in all cases, differences were observed in the levels of produced products. Further kinetic assays will be needed in order to investigate the reason for these differences in activity.

### Amy1 confers the ability to grow on starch when heterologously expressed in a *B. breve* UCC2003 ApuB-deficient mutant

In order to validate the predicted starch-degrading activity of the product of *amy1*, as its activity was not obvious from the previous enzymatic assays, the gene was cloned under the control of a constitutive promoter (see Materials and Methods), resulting in plasmid pBC1.2:*amy1*. Plasmid pBC1.2:*amy1* was then introduced in *B. breve* UCC2003:ΔApuBP8, which is a derivative of UCC2003 in which the *apuB* gene had been interrupted by transposon mutagenesis, thus making this strain unable to grow in starch or starch-like glycans (O’Connell Motherway et al., 2008; Shiver et al., 2021, 2023).

**Figure 6:**
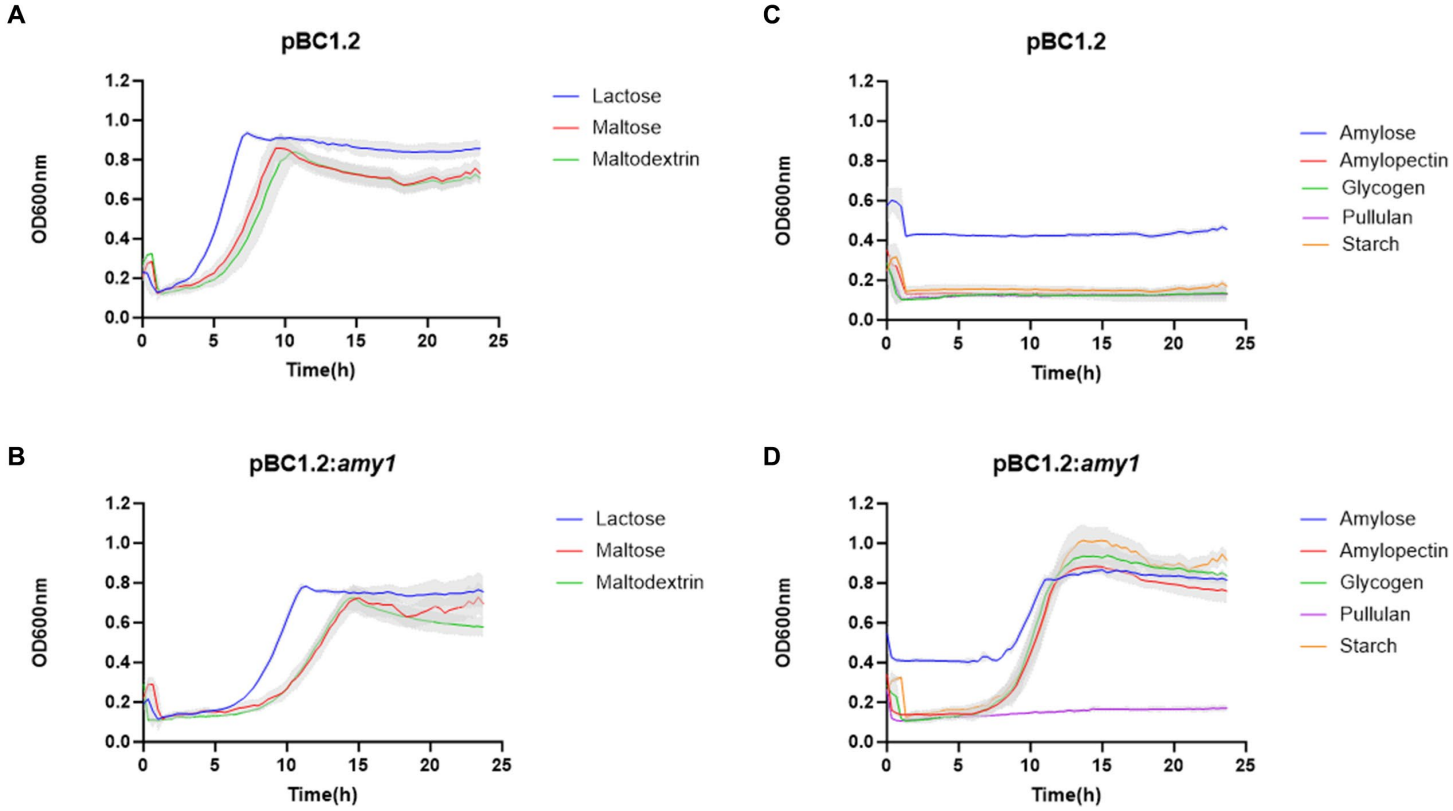
Growth profile of *B. breve* UCC2003: ApuB mutant containing pBC1.2 empty **(A,C)** and pBC1.2:Amy1 **(B,D)**. Growth was assessed in new modified Rogosa medium supplemented with 0.5% (w/v) of each of the carbohydrates specified on the legend. Graph shows the average of three independent experiments.

As observed from Figure 6, heterologous expression of *amy1* in strain *B. breve* UCC2003:ΔApuBP8 allows this strain to grow on starch, amylose, amylopectin and glycogen. It is interesting to note that the complemented mutant was unable to grow in pullulan.

### Distribution of GH-encoding genes involved in xylan and starch metabolism in members of the *B. pseudocatenulatum* taxon

To assess the prevalence of the here identified key genes involved in xylan and starch metabolism in *B. pseudocatenulatum*, we performed a blastp-based analysis to assess the presence of these key genes in all publicly available genomes of this species. For this, we used the deduced amino acid sequences of *xylB_78_* and *xylA_213_* as representatives of the GH10 xylanase responsible for xylan degradation by *B. pseudocatenulatum*; and the protein sequences of the three α-amylase-encoding genes *amy1_150_*, *amy2_150_* and *amy3_150_* of *B. pseudocatenulatum* MB0150 as key enzymes involved in starch degradation.

**Figure 7:**
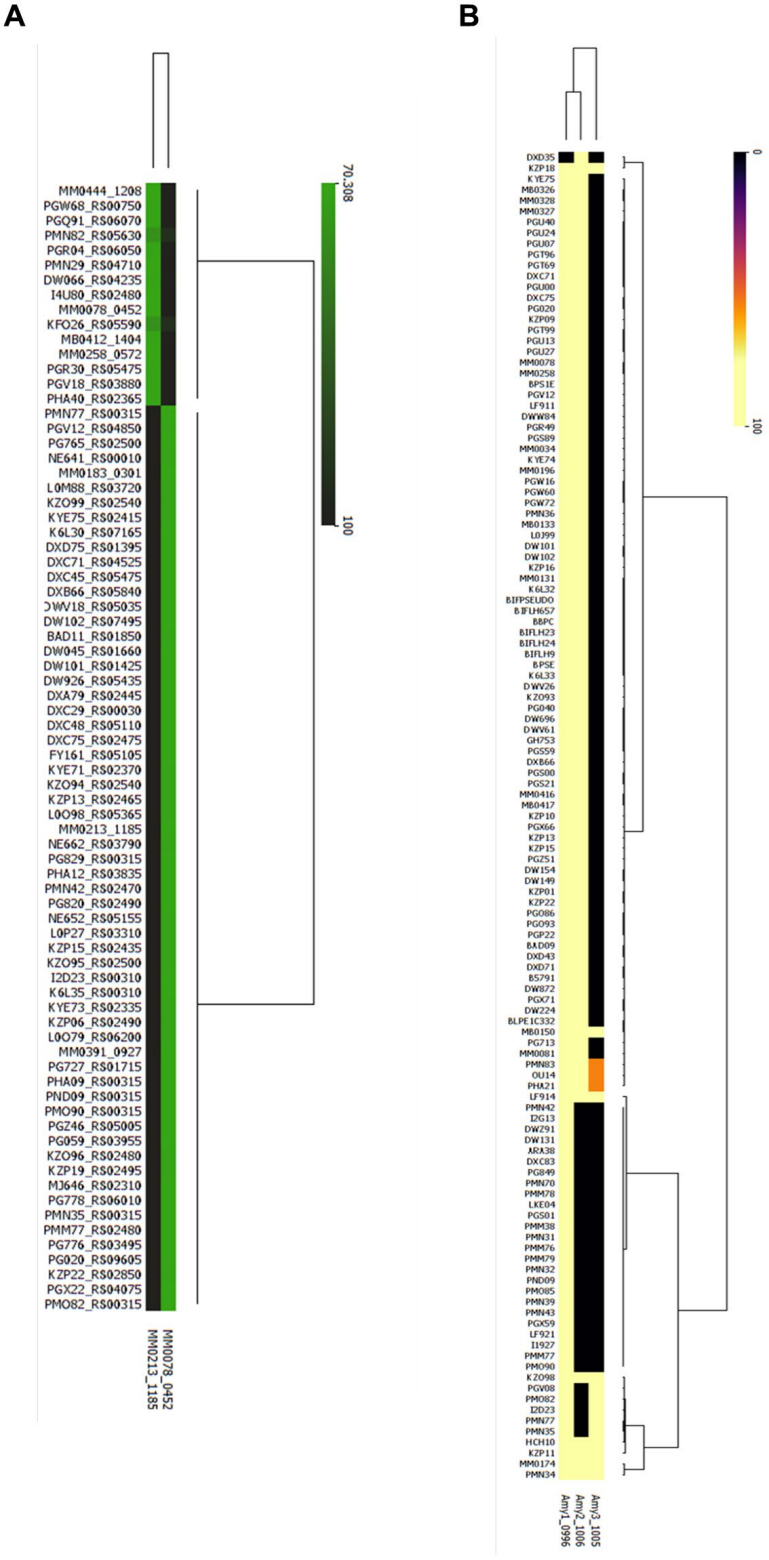
Presence/ Absence of Glucosyl hydrolases involved in xylan and starch metabolism in all publicly available genomes of *B. pseudocatenulatum*. **(A)** Heatmap representing the percentage of identity elicited in the strains where at least one endo-xylanase was identified, cutoff stablished at a 70% of identity as result of blastp **(B)** Heatmap representing the percentage of identity where at least one α-amylase was identified (Yellow: presence, black: absence).

The results of this analysis are schematically displayed in Figure 7A, revealing that 72 strains (28.6%) of the 252 publicly available *B. pseudocatenulatum* genomes possess a xylanase-encoding homologue of either XylB or XylA. The xylanase-encoding gene (*xylA* homolog) described by Watanabe et al. was the most prevalent with a total of 59 genomes encompassing a clear XylA homologue (identity >99.75%), while clear homologues of *xylB* (identity >99.48%) were determined to be present in just 13 genomes. It is worth noting that none of the assessed genomes contained both *xylA* and *xylB*.

It was observed that α-amylase-encoding genes are more prevalent among *B. pseudocatenulatum* genomes as at least two α-amylase-encoding gene were present in 109 strains or 43.3% of the 252 genomes that were scrutinized (Figure 7B). Except for one strain DXD35, where only a gene encoding Amy2 was identified, however this could be due to incomplete genome sequence data. The *amy1* and *amy2* were both shown to be present in 93 strains, while the *amy1* and *amy3* gene combination is present in just five strains. Interestingly *amy2* and *amy3* are not present together unless *amy1* is also encoded in that genome. Finally, we found that 11 publicly available genomes harbour all three described α-amylase-encoding genes. The least prevalent α-amylase-encoding gene is *amy3*, being identified in 14 strains and always being present together with a second α-amylase-encoding gene.

## Discussion

**Figure 8:**
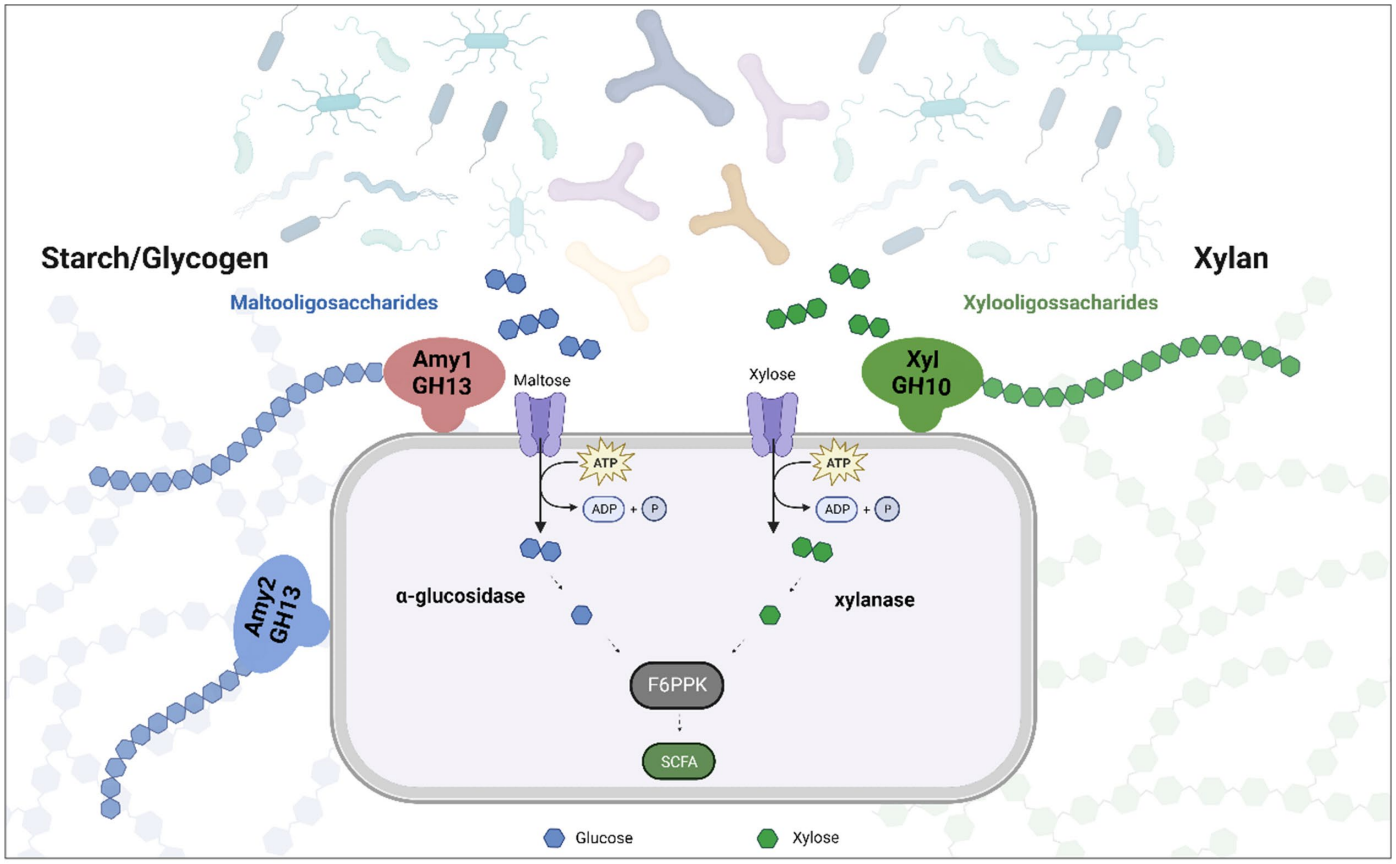
Representation of the role that play extracellular enzymes in the degradation of xylan and starch in *B. pseudocatenulatum.* Oligosaccharides released by Amy1, Amy2 and XylA allow other (bifido)bacteria to reach these substrates, promoting cross feeding.

The gut microbiota plays a key role in the degradation of dietary fibre. Various members of the *Bifidobacterium* genus are able to degrade these complex carbohydrates and contribute to the modulation of bacterial communities in the gut. Typically, adult-associated species such as *B. longum* and *B. adolescentis*, tend to have a wide range of genomic adaptations for the cleavage and degradation of dietary polysaccharides (Arboleya et al., 2018). Recently *B. pseudocatenulatum* has been postulated as a bifidobacterial species whose metabolic abilities may facilitate its adaptation to the human gut (Drey et al., 2022; Larke et al., 2023). The lack of studies focusing on this species meant that until recently this potential had not been explored. However, we show here that certain members of the *B. pseudocatenulatum* taxon are able to metabolize dietary glycans, such as xylan, starch, amylopectin, maltodextrin and glycogen, although this ability is not a conserved trait within this bifidobacterial species (Figure 8).

It has previously been established that the ability of *B. pseudocatenulatum* to digest xylan depends on an extracellular GH10 family endoxylanase enzyme (Watanabe et al., 2021). Our work shows that there are two distinct GH10 endo-xylanases encoded by different strains of *B. pseudocatenulatum*: homologs of BpXyn10A, described by Watanabe and colleagues and homologs of XylB_78_, which is encoded by strain MM0078. It is possible that these two GH10 enzymes have different preferences or exhibit a different degradation profile when utilizing other substrates, such as arabinoxylan, as has been observed for *B. longum* (Komeno et al., 2022). Arabinoxylan from cereal sources is composed of a xylan backbone with arabinose substitutions, while arabinoxylan from corn is even more complex with further substitutions (Saito et al., 2020). Just 28.6% of the assessed *B. pseudocatenulatum* genomes encodes a single GH10 family endoxylanase. This is in contrast to other members of the gut microbiome, such as *Bacteroides intestinalis*, which encodes several paralogs belonging to the GH10 family (Wang et al., 2016). These findings are therefore consistent with the notion that the predominant xylan-degrading organisms from the human colon are Bacteroidetes and Bacillota members (Zhao et al., 2023).

Through transcriptomic analysis we identified a cluster involved in starch degradation in the commensal *B. pseudocatenulatum* MM0196. Transcription of two α-amylase-encoding genes *amy1* and *amy2* was shown to be upregulated when the strain was grown in starch. A previous study in *B. adolescentis* had identified several loci, interestingly this study also identified pili formation genes overexpressed when the strain was grown in starch (Duranti et al., 2014).

A comparative analysis carried out with the set of strains from this study allowed the identification of a third α-amylase-encoding gene, *amy3_150_*, present in strain MB0150 and with a clear homolog present in strain MM0174. The predicted function of these three genes was determined by heterologous expression and enzymatic assays in a range of starch-related carbohydrates. Our results show that Amy1, Amy2 and Amy3 all exhibit α-amylase activity, and that they degrade all tested substrates that contain α-1,4-glucosidic linkages, with the exception of maltose and pullulan. A similar α-amylase, named BiLA, has previously been identified and characterized in *B. longum* by Hye-Won and colleagues, although this particular enzyme was shown to cleave pullulan, as well as amylose, amylopectin, starch and glycogen (Lee et al., 2016). This is similar to ApuB, an extracellular α-amylase encoded by *B. breve* UCC2003 (O’Connell Motherway et al., 2008).

Interestingly, the identified *B. pseudocatenulatum amy* cluster encompasses 8 genes predicted to be involved in EPS production, suggesting that the simultaneous expression of these two functions, when starch or starch-related carbohydrate are present, are linked. Possibly the presence of starch in the lumen causes the induction of EPS production in these strains, perhaps as a carbohydrate-specific signal to initiate a microbe-host dialogue mediated by EPS (Fanning et al., 2012; Castro-Bravo et al., 2018).

It is noteworthy that starch metabolism as a trait appears more conserved among members of the *B. pseudocatenulatum* taxon when compared to the ability to metabolize xylan. Both abilities provide a means through which (the degradation products of) these glycans become available to other members of the gut microbiota by means of cross-feeding, facilitating the survival of (bifido)bacteria that do not possess the ability to directly degrade these complex carbohydrates (Fernandez-Julia et al., 2022; Culp and Goodman, 2023).

Overall, this study provides insights into particular plant-glycan metabolic abilities encoded by members of the *B. pseudocatenulatum* taxon, expanding our knowledge on xylan and starch metabolism, the latter of which had not previously been studied in this species. This study did not characterize transporters involved in the internalization of starch/xylan degradation products, and further studies similar to that by Saito and colleagues would be needed to fully understand the associated metabolic patwhays (Saito et al., 2020). The identification and characterization of the GH enzymes involved in the initiating step of starch and xylan degradation contribute to our understanding of the role that certain strains of this taxon play in starch/(arabino)xylan-based self- and cross-feeding, thereby potentially strengthening gut microbiota homeostasis with possible positive implications on human health.

## Materials and methods

### Bacterial strains, plasmids and culture conditions

*B. pseudocatenulatum* strains were routinely cultured under anaerobic conditions at 37°C in modified de Man Rogosa Sharpe (mMRS) medium prepared from first principles and supplemented with 0.5% lactose and 0.06% cysteine-HCl (Watson et al., 2013). *B. pseudocatenulatum* cultures were cultivated under anaerobic conditions in a modular atmosphere-controlled system (Davidson and Hardy, Belfast, Ireland) at 37°C. *Escherichia coli* strains were cultivated in LB broth (Oxoid Ltd., Basingstoke, England) at 37°C.

### Growth assessment

Growth profiles of *B. pseudocatenulatum* strains were determined manually in mMRS Rogosa using 0.5% (w/v) of Lactose, Starch, Glycogen, Maltodextrin, Amylopectin, Amylose, Arabinoxylan, Xylan as a sole carbon source. 5 mL of media supplemented with 0.5% (w/v) of a given carbohydrate and 0.06% (w/v) of Cysteine-HCl was inoculated with 100 μL of an overnight culture normalized to an OD_600nm_ of ~2. All cultures were cultivated under anaerobic conditions during 10 or 24 h.

### Nucleotide sequence analysis

Sequence data were obtained from Artemis-mediated genome annotations of *B. pseudocatenulatum* strains. Database searches were performed using non-redundant sequences accessible at the National Centre for Biotechnology Information (http://www.ncbi.nlm.nih.gov) using BLAST. Sequences were verified and analyzed using Benchling (United States).

### DNA manipulations

Chromosomal DNA was isolated from *B. pseudocatenulatum* MB0150, MM0078 and MM0213 using 1.5 mL of an overnight culture using the Genelute bacterial genomic DNA kit (Sigma). Plasmid DNA using the GeneJET Plasmid Miniprep Kit (Thermo Scientific, UK). All restriction enzymes and T4 DNA ligase were used according to the supplier’s instructions (New England Biolabs, UK). Synthetic single stranded oligonucleotide primers used, specified in Table 2, were synthesized by Eurofins (Ebersberg, Germany). Standard PCRs were performed using Dreamtaq (Thermo Scientific, USA). PCR products were visualized by Greensafe following agarose gel electrophoresis (1% agarose). PCR fragments were purified using the Roche high Pure PCR purification kit (Roche Diagnostics, Basel, Switzerland). Plasmid DNA was isolated using GeneJET Plasmid Miniprep Kit (Thermo Scientific, UK) and transformed into *E. coli* BL21 by electroporation according to published protocols.

**Table 2:** Primers used in this study.

Primer name	Sequence (5′-3′)	Description
0996_HindIII_Fw	TGCATC**TCTAGA***TTGACAGCTAGCTCAGTCCTAGGTATAATGCTAGC*gatgcaaggaagcaggag	To amplify the gene 0996
0996_XbaI_Rv	TGCCTC**AAGCTT**CAAAGCACGCCCTTATAC	To amplify the gene 0996
PET_0996F_NheI	TGCATC**GCTAGC**gagagcatgaagaacggc	To amplify the gene 0996
PET_0996R_XhoI	TGCCTC**CTCGAG**cggtggatgtatggcttt	To amplify the gene 0996
PET_1,005_Fw_NheI	TGCATC**GCTAGC**agcaccgaccgtaacagtta	To amplify the gene 1,005
PET_1,005_Rv_BamHI	TGCCTC**GGATCC**gggctatttcacttgacgg	To amplify the gene 1,005
PET_1,006_Fw_NheI	TGCATC**GCTAGC**gccacccgagacagctatg	To amplify the gene 1,006
PET_1,006_Rv_XhoI	TGCCTC**CTCGAG**gccgtttacttgacggtg	To amplify the gene 1,006
PET_78_0452_Fw_NheI	CATTCA**GCTAGC**gactatccgggtggcatc	To amplify domain 0472
PET_78_0452_Rv_XhoI	CATTCA**CTCGAG**tcaagtctgcggcatcttgtc	To amplify domain 0472
PET_213_1,185_Fw_NheI	CATTCA**GCTAGC**gactactccggtggcatcaag	To amplify domain 1,185
PET_213_1,185_Rv_XhoI	CATTCA**CTCGAG**tcaggtctgcggcatcttgtc	To amplify domain 1,185

^*^Restriction sites incorporated into oligonucleotide primer sequences are indicated in bold and promoted region incorporated into nucleotide primer sequences are indicated in italics.

### RNAseq analysis

*B. pseudocatenulatum* MM0196 was grown in lactose or starch as the sole carbon source (0.5%) in New modified Rogosa media supplemented with 0.06% Cysteine-HCl until they reached an OD_600nm_ of ~0.5. 5 mL of the culture was centrifuged at 5500 rpm at 4°C and immediately resuspended in 500 μL of Zymo RNA shield Solution 2X and stored at -80°C. Triplicate of the samples were prepared. The cell pellets were sent in dry ice to BaseClear (Leiden, Belgium) that performed the RNA extraction using ZymoBIOMICS™ RNA Miniprep Kit (Zymo R2001) extraction kit and RNA sequencing using Novaseq 6,000 (Illumina).

### Construction of overexpression vectors

For the construction of recombinant plasmids, various DNA fragments containing the targeted genes were amplified with Q5 High Fidelity polymerase (New England Biolab, UK) using chromosomal DNA extracted with Genelute bacterial genomic DNA kit (Sigma) as a template and employing primer combinations as specified in Table 2. The amplicon encompassing *amy1_150_* (corresponding to locus tag MB0150_0996) was digested with HindIII and XbaI, while the amplicon containing *amy2_150_* (locus tag MB0150_1,005) with XbaI and BamHI; and amplicon containing *amy3_150_* (locus tag MB0150_1,006) with XbaI and XhoI. Plasmid pBC1.2 was digested with the same enzyme combinations as used for the corresponding, to be cloned amplicons. The *amy1_150_*, *amy2_150_* and *amy3_150_* genes were also amplified using different primers (see Table 2) so as to exclude the signal sequence (predicted by means of SignalP 5.0), for the heterologous expression in pET28b. In this case different enzymes were used to digest the amplicon, *amy2_150_* was digested with NheI and BamHI, while amplicons encompassing *amy1150*, *amy3_150_*, *xylA_78_* (locus tag MM0078_0452) and *xylB_213_* (locus tag MM0213_1,185) were digested with NheI and Xhol (All enzymes were obtained from New England Biolab, UK).

All PCR products and their corresponding plasmids were ligated using T4 ligase (New England Biolabs, UK). The ligation mix of the pBC1.2 constructs was introduced by electroporation into *E. coli* EC101 and transformants were selected with 10 μg/mL chloramphenicol.

The ligation corresponding to the pET28b constructs was introduced by electroporation into *E coli* BL21 and transformants were selected using 50 μg/mL kanamycin. Transformants were screened by plasmid extraction and digestion analysis as well as PCR using primers described on Table 2. All constructs were verified by sequencing (SNPsaurus, US).

### Protein overproduction and purification

250 mL cultures of recombinant *E. coli* strains were grown in NZY Auto-Induction LB medium (NZYtech) for 24 h at 24°C. Cells were harvested by centrifugation at 4°C, for 10 min at 5500 rpm. Mechanical lysis was performed using a bead beater for a total of 3 min (with 1 min of incubation on ice following every minute of bead beating) and supernatants were collected after a centrifugation step of 20 min at 10,000 rpm, at 4°C. Protein purification was performed using His-tag affinity gravity-flow chromatography, using Ni-NTA matrices in accordance with the manufacturer’s instructions (Qiagen).

### Enzymatic reactions

The purified His-tagged proteins were dialysed and concentrated using Amicon Ultra-4 4ML – 10 kDa by combining all elution aliquots and washing with 10 mL of 20 mM MOPS buffer adjusted to pH 7. The protein concentration was assessed using Qubit using manufacturer instructions. Reactions were prepared in a volume of 1 mL and using an initial concentration of 1 mg/mL of substrate (Starch, Glycogen, Maltodextrin, Amylopectin, Amylose) and adding 10 μg of protein. After 24 h incubation at 37°C, the reactions were inactivated by incubating them at 85°C for 15 min and then used for HPAEC-PAD analysis to assess possible degradation products (see Table 3).

**Table 3:** Plasmids and strains used for cloning in this study.

	Description	Reference
*Strains*		
*Escherichia coli* BL21	Cloning host	Commercially available
*E. coli* EC101 *Bifidobacterium breve* UCC2003	Cloning host Cloning host	Commercially available O’Connell Motherway et al. (2011)
*Bifidobacterium breve* UCC2003:ΔApuB	Cloning host	Shiver et al. (2021), Shiver et al. (2023)
*Plasmids*		
pET28.b	Km^r^, cloning vector carrying IPTG inducible promoter	Commercially available
pET28b:0452	Km^r^; containing x*ylA*	This study
pET28b:1185	Km^r^; containing x*ylB*	This study
pET28b:0996	Km^r^; containing a*my1*	This study
pET28b:1005	Km^r^; containing a*my2*	This study
pET28b:1006	Km^r^; containing a*my3*	This study
pBC1.2:amy1	Cm^r^; containing a*my1*	This study

### HPAEC-PAD analysis

For HPAEC-PAD analysis, a Dionex (Sunnyvale, CA) ICS-6000 system was used. Carbohydrate fractions from the above-mentioned hydrolysis assays (25 μL aliquots) were separated on a CarboPac PA1 analytical-exchange column (dimensions, 250 mm by 4 mm) with a CarboPac PA1 guard column (dimensions, 50 mm by 4 mm) and a pulsed electrochemical detector (ED40) in PAD mode (Dionex). Elution was performed at a constant flowrate of 0.063 mL/min at 30°C. The following linear gradient of potassium hydroxide was used with 10 mM KOH: from 0 to 8 min, 10 mM; from 8 to 18 min, 10–40 mM; from 18 to 5 min, 40 mM; from 25 to 30 min, 10 mM. Chromatographic profiles of standard carbohydrates were used for comparison of the results of their breakdown by Amy1_His_, Amy2_His_, Amy3_His_, XylB_His_ and XylA_His_ proteins. Chromeleon software (version 7; Dionex Corporation) was used for evaluation of the obtained chromatograms. A 1 mg/mL stock solution of each carbohydrate, as well as their putative breakdown products (where available) used as reference standards was prepared by dissolving the sugar in Milli-Q water.

## Data availability statement

The RNAseq data presented in this study are deposited in the NCBI repository under the Bioproject accession number PRJNA1120230.

## Ethics statement

Ethical approval for the study was received from National Maternity Hospital research ethics committee in February 2016 (EC 35.2015). The studies were conducted in accordance with the local legislation and institutional requirements. Written informed consent for participation in this study was provided by the participants’ legal guardians/next of kin.

## Author contributions

RS-G: Conceptualization, Data curation, Investigation, Methodology, Validation, Visualization, Writing – original draft, Writing – review & editing, Formal analysis. FB: Software, Writing – review & editing. LF: Investigation, Writing – review & editing. ME-T: Methodology, Validation, Writing – review & editing. CS: Investigation, Writing – review & editing. RM: Writing – review & editing, Methodology. FM: Funding acquisition, Writing – review & editing. PC: Funding acquisition, Supervision, Writing – review & editing. DS: Conceptualization, Funding acquisition, Resources, Supervision, Writing – review & editing, Project administration.
