# Supplementary material for: Unveiling metabolic pathways of selected plant-derived glycans by Bifidobacterium pseudocatenulatum
Source: Front Microbiol. 2024 Jul 16;15:1414471. doi: 10.3389/fmicb.2024.1414471 (PMC11286577; doi:10.3389/fmicb.2024.1414471)
Supplement: Supplementary file 1 [file Presentation_1.pptx]

## Slide 1
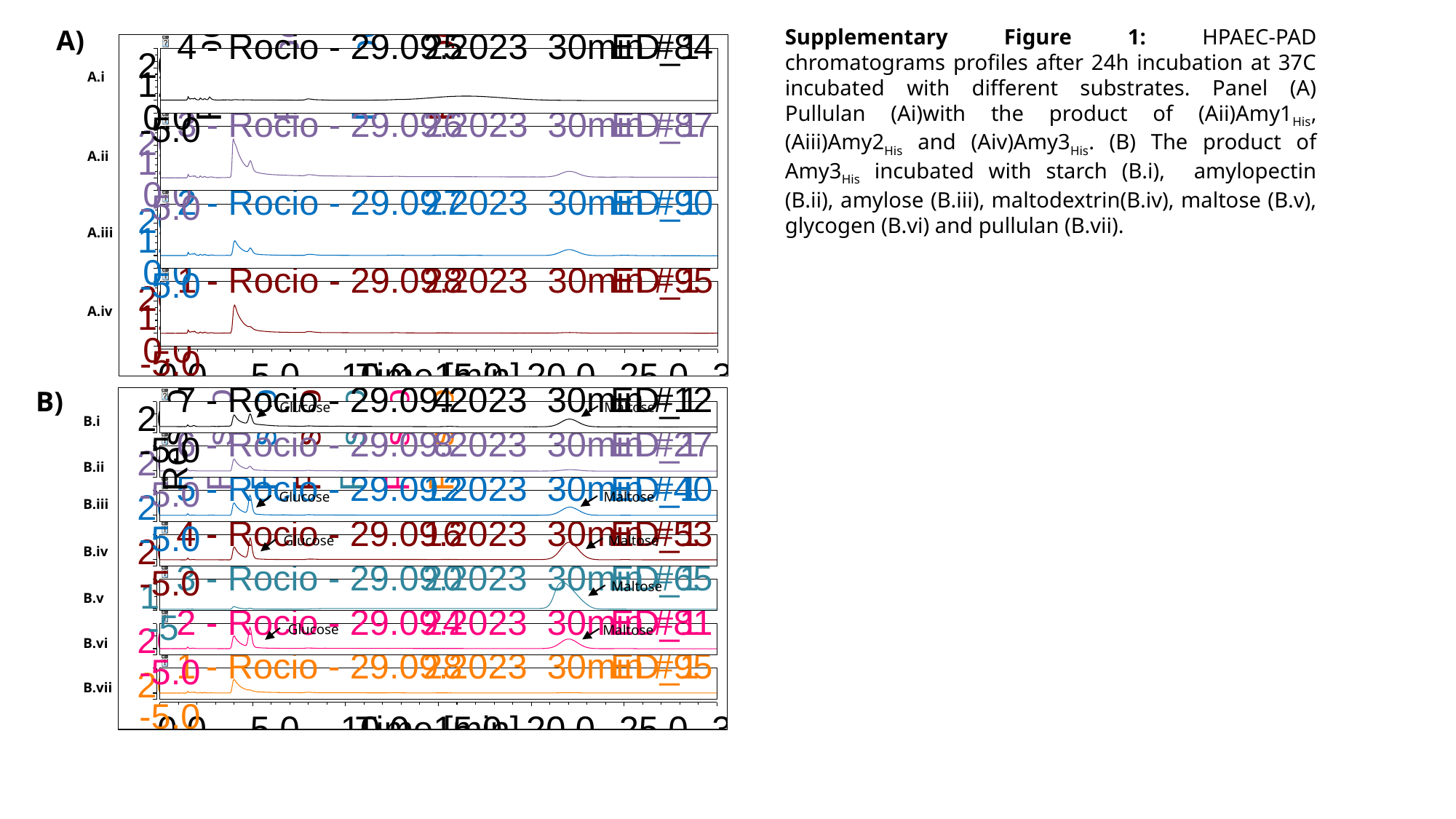

A)
Supplementary Figure 1: HPAEC-PAD chromatograms profiles after 24h incubation at 37C incubated with different substrates. Panel (A) Pullulan (Ai)with the product of (Aii)Amy1His, (Aiii)Amy2His and (Aiv)Amy3His. (B) The product of Amy3His incubated with starch (B.i), amylopectin (B.ii), amylose (B.iii), maltodextrin(B.iv), maltose (B.v), glycogen (B.vi) and pullulan (B.vii).
A.i
A.ii
A.iii
A.iv
B)
Glucose
Maltose
B.i
B.ii
Glucose
Maltose
B.iii
Glucose
Maltose
B.iv
Maltose
B.v
Glucose
Maltose
B.vi
B.vii
